# Supplementary material for: Attention 3D central difference convolutional dense network for hyperspectral image classification
Source: PLoS One. 2024 Apr 10;19(4):e0300013. doi: 10.1371/journal.pone.0300013 (PMC11006129; doi:10.1371/journal.pone.0300013)
Supplement: Attachment — Submitted filename: R2_Reply_to_reviewer__PONE_D_23_32380R1_3D_CNN.pdf [file pone.0300013.s003.pdf]

Manuscript ID: PONE-D-23-32380R1

Article title: Attention 3D Central Difference Convolutional Dense Network for Hyperspectral Image Classification

February 14, 2024

Dear Academic Editor,

First of all, we would like to extend our sincere gratitude to Academic Editor, for allowing a re-submission of our manuscript, with an opportunity to address the reviewers' comments. We greatly appreciate the Reviewers' time spent reviewing the paper and assisting in its improvement. These comments are very helpful for revising and improving the paper, as well as the important guiding significance to the other research. We have studied the comments very carefully and made the corrections that we hope to meet with the final approval. We have done our best to modify the paper to reflect all the comments made by the Academic editor and anonymous reviewers. These changes are fully addressed in this reply letter.

Below, we provide point to point responses explaining how we have addressed each of the Reviewer's comments. For clarity, our responses have been written in blue color.

Reviewer 1: Note; Most of your comments are revised, which were addressed in the first revision. In this revision we have refined them, hopefully, this will fulfill the requirements. These revisions are highlighted in magenta color.

Concern # 1

This article lacks a background introduction to such massive data. Writing background refers to the historical background in which the author created this article and the context in which it was created. Therefore, please optimize the writing background of the article so that it can have a deeper understanding of the content.

Respected reviewer,
We sincerely thank you for guiding us to enrich the manuscript's content. The revisions made have significantly bolstered the depth and relevance of the literature review, ensuring that it adequately reflects the advancements in the field. In the revised version we have included the related work section

Related work

Recently, deep learning techniques for hyperspectral image (HSI) classification have been extensively explored. Various methods like Stacked Autoencoders (SAEs) [18], Deep Belief Networks (DBNs) [2], Deep Boltzmann Machines (DBMs) [20], and Convolutional Neural Networks (CNNs) [4,10,13,16,25,26] have garnered significant attention. SAE [18], for instance, functions as an unsupervised feature extraction technique by sequentially stacking autoencoders, allowing for the extraction of spectral-spatial features.

CNNs have seen widespread use in HSI classification. For instance, a basic CNN model with five layers was proposed [10], focusing solely on spectral information. To address this limitation, an enhanced CNN model [26] was introduced, utilizing 3-D patches as input to incorporate both spectral and spatial information. Furthermore, another approach involves a CNN integrated with a spatial pyramid pooling strategy to contain spatial information [26] comprehensively. Additionally, there's a proposition that combines CNN features with hand-crafted features and Conditional Random Field (CRF) [22]. Another variant, CNN with Markov Random Field (MRF) [4], was introduced to leverage label correlations effectively.

A dual-channel CNN [27] was introduced, utilizing 1-D and 2-D CNNs for feature extraction. To expand the training dataset for deep CNNs, a novel pixel-pair method [16] was proposed. Moreover, a 3-D Convolutional Neural Network (3D-CNN) [7] was introduced, enabling joint spectral and spatial information processing. Similarly, a 3-D Contextual deep CNN (3D-FCN) [13] was suggested to optimize the exploration of local contextual interactions among neighboring individual pixel vectors. The advancement of Convolutional Neural Networks (CNNs) has spurred the development of various convolution techniques. One such technique, tiled convolution, employs distinct filters for feature map neurons with nearby receptive fields on the input image [21]. Consequently, this method generates a feature map using multiple filters, extracting more definitive features with an equal number of feature maps. In a study, the augmented linear mixing model (ALMM) tackles spectral variability in hyperspectral images by using a data-driven approach to isolate scaling factors linked to illumination or typography using an end-member dictionary, while also capturing additional variations from environmental factors and instrument settings via spectral variability dictionary [8]. Their proposed method, integrated into the spectral unmixing framework, allows for the concurrent acquisition of the spectral variability dictionary and the estimation of abundance maps, showcasing enhanced effectiveness compared to earlier advanced techniques in experiments conducted on both synthetic and real datasets.

Dilated convolution [26], another convolutional approach, focuses on broadening the receptive field of a filter without increasing the parameters. This is achieved by

introducing cells with zero weight values into the filters. Studies have indicated that this technique may enhance performance in certain scenarios [26].

In a different study, micro Multi-Layer Perceptron (MLP) structures are utilized as filters, referred to as Network In Network (NIN) [17]. This enables the filters to learn more intricate relationships during the training phase. Another notable alternative convolution technique is Inception [24], where varying-sized filters are incorporated within a single convolution layer. This method can simultaneously execute convolution and pooling processes, demonstrating improved performance without escalating the parameter count by utilizing the inception module. While deep learning methods have accomplished impressive performance in HSI classification, they often require more data as input. Moreover, unlike traditional descriptors, Convolutional Neural Networks (CNNs) tend to overfit easily and face challenges in generalizing well to unseen scenes. This difficulty in generalization can pose a significant issue when applying CNNs for HSIs, hindering their adaptability to diverse and unfamiliar environments or scenes. Additionally, the reliance of these methods on extensive sequences as input poses practical limitations, particularly in scenarios where real-time processing is necessary. Therefore, despite achieving state-of-the-art results, these drawbacks highlight the need to improve the generalization ability and adaptability of CNN-based approaches in HSI classification tasks. The convolution operator plays a vital role in extracting fundamental visual features within the deep learning framework.

Recently, there have been developments and extensions to the conventional convolution operator. One direction involves incorporating classical local descriptors like LBP [15] and Gabor filters [3] into convolution design. Notable works include Local Binary Convolution [11] and Gabor Convolution [19]. These innovations aim to save computational resources and enhance resistance to spatial changes. For instance, Local Binary Convolution is devised to reduce computational costs, while Gabor Convolution aims to improve resilience against spatial alterations. Another direction in extending convolution operators involves modifying the spatial scope for aggregation. Noteworthy works in this area include dilated convolution [5] and deformable convolution [28]. These adaptations aim to alter the convolution's receptive field, allowing for wider spatial information aggregation.

However, these convolutional operators have primarily been designed and studied for the RGB modality. How effectively they perform when applied to depth and abundant spectral modalities remains uncertain. Understanding their performance across different modalities, such as depth and Hyperspectral data, requires further exploration and investigation. Specifically, how these modified convolutional techniques function and adapt to HSI data is an open question that must be addressed to understand their efficacy across various modalities comprehensively. These alternative techniques aim to increase the number of acquired features from a sole convolution layer. Aligning with this notion, this paper introduces a novel convolution technique

termed " central difference convolution." The proposed method harbors unique qualities and advantages compared to existing approaches.

Concern # 2

In the introduction section of the article, the author's expression of the writing meaning is somewhat vague and difficult to understand. Please reorganize the language to express it so that readers can quickly understand the writing meaning and purpose of the article.

Respected reviewer,
Thank you for your valuable suggestion to enhance the introduction section. We've revised it by simplifying the language and replacing complex words with simpler ones. As a result, the updated introduction is now clearer and more easily understandable. major changes are highlighted, with color

Concern # 3

Provide information on the specific data used in your study. Mention the source and characteristics of the data.

Respected reviewer,.
we appreciate your concern related to the dataset used in our study, we have provided more detail regarding to datasets and also mentioned the source, please see the detail from line number 336-374.

Datasets Explanation

During the experimental phase of this study, the analysis included three Hyperspectral datasets: Indian-Pines (IP), University of Pavia (UP), and Houston-2018 (HT). Additionally, a comprehensive description of each Hyperspectral dataset was provided. These datasets are publicly available for the experiments and can be downloaded from the website www.ehu.eus [23] **Indian Pines (IP):** In 1992, the Air-Borne Visible/Infrared Imaging Spectro-meter [6] sensor was utilized to procure the dataset recognized as IP. The area comprised several agricultural fields with an organized geometric structure and some areas of irregular forest. The image under analysis encompasses a vast $(145 \times 145)_P$ pixel array, containing an extensive collection of 224 spectral bands that span the wavelength range of 400 to 2500 nanometers, all of which are captured at a remarkable spatial resolution of 20 meters per pixel. After removing four null bands and 20 other bands affected by atmospheric water absorption, the pre-processed data consisted of 200 remaining bands utilized for experimentation. Additionally, almost 50% of the dataset, i.e., $(10,249)_P$ pixels out of the total $(21,025)_P$, contained ground-truth information that provided a single label belonging

to one of the 16 different classes.

Pavia University (UP): The UP dataset, including the Northern Italian campus of UP, was obtained using the Reflective Optics System Imaging Spectro-meter [12] sensor. It mainly encompasses an urban environment characterized by numerous solid structures such as Asphalt, Gravel, and metal sheets, along with natural objects such as Trees, Meadows, and Soil. The dataset also includes shadows. After the elimination of the noisy bands, a total of 103 spectral bands were obtained within the spectral range of 0.43 to 0.86 meter, with a spatial resolution of $(1.3)_{MPP}$, and consisting of $(610 \times 340)_P$ pixels in size, each matched pixel by pixel. Out of the total of $(207, 400)_P$ pixels, a significant proportion comprising precisely 20%, amounting to $(42, 776)_P$ pixels, have been accurately annotated to contain authentic ground-truth information belonging to as many as nine distinctive class labels. The present circumstance entails the utilization of a model processing size measuring 200 units in width, 200 units in height, and 103 units in bands.

Houston 2018 (HT): The present study involves the analysis of a scene from Houston in 2018, which encompasses an area of 210 by 954 pixels, comprising a total of 48 spectral-bands within the wavelength range of 380 to 1050 nanometers, with 1 ground sample interval meter. The ground-truth data for the Houston 2018 scene has a pixel size of 0.5, meaning each pixel in the image corresponds to a physical area of 0.5 square units. To process the Houston 2018 scene, a model with dimensions of 200 units in width, 200 units in height, and 48 units in bands is utilized.

Concern # 4

Describe the simulation environment and parameters used for testing the algorithm.

Respected reviewer,
Thank you for your valuable feedback regarding the simulation environment and parameters utilized in our study. In response, we have implemented a dedicated section titled "Experimental Settings," wherein we comprehensively list and detail all the training and testing parameters employed in our experiments.

Experimental Settings

Experiments are done in this study using the proposed model and comparing methods. Three well-known HS HSI datasets were taken to check the efficiency of the 3DCDCN. Houston-2018 (HT), IP, and PU were used to test. All the experiments were done in Colab Pro. In all experiments, the initial Test/Train set was divided into a 30 : 70, ratio, with 70% of the population reserved for train samples and the remaining 30% for test samples. To ensure fair evaluations, we used a standardized learning rate of

(1$e-02$) and a decay rate of (1$e-06$) for all experiments. Furthermore, we utilized the rectified linear unit activation function in all layers except for the last layer, where we employed the softmax activation. The patch sizes used in our experiments were $13 \times 13 \times 20, 17 \times 17 \times 20, 21 \times 21 \times 20$, and $25 \times 25 \times 20$, respectively. These patch sizes were determined using the PCA method to identify the 20 most informative bands. For optimization, the Adam optimizer was used, and 100 epochs were set to train the model.

Concern # 5

Include a discussion on the metrics used to evaluate the algorithm's performance and why these metrics were chosen.

Respected reviewer,
We appreciate your suggestion regarding the inclusion of a discussion on the evaluation metrics used to assess the algorithm's performance. In the updated version, we've incorporated a detailed section discussing the chosen evaluation metrics and the rationale behind their selection.

Evaluation Metrics

The evaluation of hyperspectral image (HSI) classification performance requires the use of multiple assessment metrics, namely overall accuracy (OA), average accuracy (AA), and kappa coefficient (Kappa), which are interconnected and intricate. OA measures the proportion of correctly classified test samples among the total testing samples, calculated by the given equation.

$$OA = \frac{\text{No. of correct predictions}(\sum_i X_{test})}{\text{Total number of predictions}} \tag{1}$$

AA calculates the average accuracy across different classes. The mathematical equation of the AA is given by

$$AA = \frac{1}{\text{No. of classes}} \sum_{i=1}^{n} \frac{\sum_{j=1} X_i^j}{T_i} \tag{2}$$

$Kappa$ is a statistical metric that quantifies the agreement between the ground truth and the classification map and is calculated by the equation.

$$k = \frac{(A_o - A_e)}{1 - A_e} \tag{3}$$

A comprehensive analysis of these evaluation measures is essential for accurately assessing HSI classification performance.

Concern # 6

Provide a more detailed presentation of the simulation results, including tables or graphs to illustrate the improvements achieved.

Respected reviewer,
Thank you for your valuable feedback regarding the presentation of simulation results. In response, we have enhanced the manuscript by providing a more detailed presentation of the simulation outcomes. We have included all window sized maps of all the models more discussion on the experiments are included in the current version and to vividly illustrate the improvements achieved by our proposed approach.

We performed extensive experiments using the proposed method and comparing methods to find the efficiency of our proposed method.

First, all the models were tested by selecting the window size 13×13. This is the small window size of our experiments. 20 number of spectral bands was used with all the window sizes. All comparing model results are listed in Table 2. The 3D-FCNN algorithm showed good results on the HT and PU datasets but could not maintain consistency on the IP dataset. It predicted an overall accuracy of 95.21%, which is less than 0.02 than the 3D-CNN model 3D-CNN provided better results on the IP dataset than the rest of the algorithms except the proposed model. In this series, Jigsaw could not show the performance on HT, PU, and IP datasets. In contrast, the proposed method showed superior results than the comparing methods and achieved OA=98.75%, AA=79.01%, Kappa=92.29% on the HT dataset, and achieved OA=99.57%, AA=89.97%, Kappa= 99.46% on PU where as scored OA=98.94%, AA=98.48%, Kappa=98.79% on the IP dataset which are the good results on this window size.

To further check the reliability of the proposed method against the comparing methods on different patch sizes, experiments were performed using the window size or patch size 17×17, where 3D-SSCNN showed satisfactory results on HT and PU dataset but provided the lower AA=71.78% which is lower than 3D-CNN and predicted the lower OA on IP, which is lower than 3D-CNN and 3D-FCNN. HYbridSN and JIgsaw-HSI predicted the overall poor numeric values. Whereas the proposed methods predicted the overall top-class results on the measuring scales, these results are listed in Table 3

When we came to the experiments with window size 21×21, The proposed 3D-CDC attention-dense net showed effectiveness and superior results. Meanwhile, Jigsaw-HSI improved the accuracy on the measuring scales but still produced the lowest prediction. 3D-FCCNN could only produce better OA (97.50%) on HT and showed unsatisfactory results on the remaining measuring scales and the as well as on the other two datasets. These outcomes are recorded in Table 4. To make the experiments more comprehensive, a 25×25 patch size was also used where 3D-FCCNN predicted OA=97.55%, which is the second highest result on HT, but AA was still lower than

most of the methods, which placed this model in the comparison line at 3rd place. Hybrid-SN and Jigsaw-HSI could not show the prominent classification prediction. On the other hand, the proposed 3D-CDCN Attention Dense Net consistently maintains the highest accuracy on all the evaluation metrics. These results can be seen in Table 5. In Figure 4, we can observe the classification maps of Houston 2018 (HT), Pavia University (PU), and Indian Pines(IP). These maps present the geographical characteristics of each class based on different window sizes (spatial dimensions).

Table 1: Based on our research findings, the efficacy of the proposed model is contingent upon the size of the window.

Datasets	HT			PU			IP		
Window size	OA%	AA%	Kappa%	OA%	AA%	Kappa%	OA%	AA%	Kappa%
13x13	97.75	76.01	92.29	99.57	87.97	99.46	98.94	98.48	98.79
17x17	97.78	74.28	92.54	99.80	99.41	99.75	98.94	99.16	98.79
21x21	97.82	73.80	92.44	99.86	99.83	99.82	98.71	98.50	98.53
25x25	97.93	71.74	92.80	99.89	88.82	99.86	99.38	99.45	99.30

Table 2: In light of the spatial dimension of the 13×13 window size, comparative evaluations were conducted with comparing methods

Datasets	HT			PU			IP		
Models	OA	AA	Kappa	OA	AA	Kappa	OA	AA	Kappa
3D-CNN	97.63	78.07	91.75	98.95	79.57	98.69	95.23	95.7	94.55
3D-SSCNN	97.26	76.20	90.93	99.44	88.19	99.30	93.03	92.66	92.04
3D-FCCNN	98.36	64.92	91.46	99.38	77.51	99.23	95.21	71.28	94.53
HYbrid-SN	95.36	47.46	84.06	99.18	77.01	98.98	76.82	50.08	72.91
JIgsaw-HSI	91.66	40.12	67.21	89.93	69.51	87.29	49.63	24.99	38.87
Proposed	**98.75**	**79.01**	**92.29**	**99.57**	**89.97**	**99.46**	**98.94%**	**98.48**	**98.79**

Table 3: In light of the spatial dimension of the 17×17 window size, comparative evaluations were conducted with cutting-edge deep learning techniques.

Datasets	HT			PU			IP		
Models	OA	AA	Kappa	OA	AA	Kappa	OA	AA	Kappa
3D-CNN	96.23	75.67	86.17	99.03	76.89	98.79	95.30	92.34	94.63
3D-SSCNN	96.73	71.78	89.28	99.57	88.32	99.46	94.67	94.54	93.91
3D-FCCNN	95.69	48.26	85.31	99.41	77.66	99.27	96.30	72.45	95.77
HYbrid-SN	96.6	57.50	88.38	98.75	75.30	98.44	74.07	43.56	69.45
JIgsaw-HSI	94.51	53.38	79.79	92.32	72.54	72.54	62.04	37.61	55.37
Proposed	**97.78**	**76.28**	**92.54**	**99.80**	**99.4**	**99.75 %**	**98.94**	**99.16**	**98.79**

Concern # 7

Discuss the computational complexity of your proposed algorithm and how it compares to existing methods.

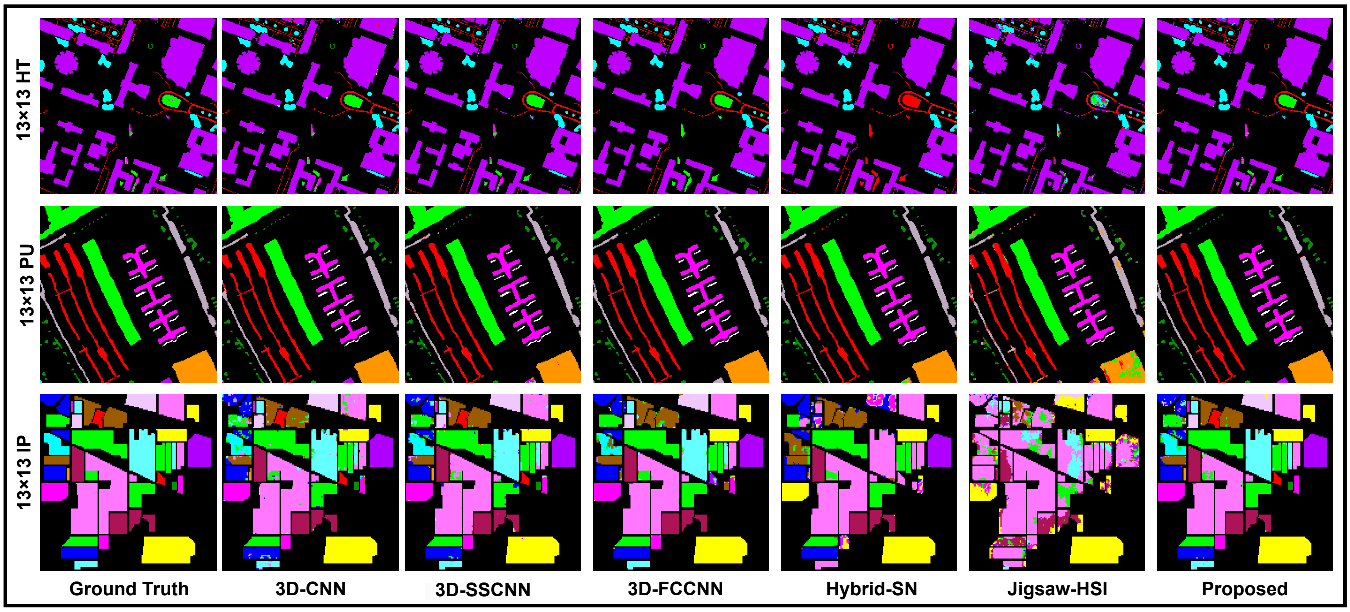

Figure 1: Our models, which incorporate state-of-the-art techniques, perform sophisticated processing of the Ground Truths with high precision and accuracy, considering the complexity of each spatial dimension.

Table 4: In light of the spatial dimension of the 21 × 21 window size, comparative evaluations were conducted with cutting-edge deep learning techniques.

Datasets	HT			PU			IP		
Models	OA	AA	Kappa	OA	AA	Kappa	OA	AA	Kappa
3D-CNN	96.77	72.80	88.36	99.17	77.57	98.96	96.36	84.74	95.84
3D-SSCNN	96.41	67.87	88.34	99.49	88.49	99.36	95.63	83.07	95.01
3D-FCCNN	97.50	59.99	91.46	99.32	77.28	99.15	97.12	73.44	96.17
HYbrid-SN	96.87	63.95	92.38	98.69	75.09	98.36	75.76	48.53	71.60
JIgsaw-HSI	95.20	58.06	82.34	95.16	76.89	93.93	73.04	51.11	68.67
Proposed	**97.82**	**73.80**	**92.44**	**99.86**	**99.83**	**99.82**	**99.89**	**88.82**	**99.86**

Table 5: Based on our research findings, the efficacy of the proposed model is contingent upon the 25 × 25 size of the window.

Datasets	HT			PU			IP		
Models	OA	AA	Kappa	OA	AA	Kappa	OA	AA	Kappa
3D-CNN	97.46	66.96	91.07	99.17	77.57	98.96	97.75	95.35	97.43
3D-SSCNN	96.56	72.01	88.84	98.86	87.70	98.58	96.62	95.06	96.14
3D-FCCNN	97.55	60.14	91.60	99.30	77.21	99.13	96.73	73.10	96.27
Hybrid-SN	95.12	46.52	83.15	96.63	65.42	95.78	79.17	51.35	75.70
Jigsaw-HSI	94.78	58.60	80.33	96.86	79.96	96.07	82.39	65.41	79.68
Proposed	**97.93**	**71.74**	**92.80**	**99.89**	**88.82**	**99.86**	**99.38**	**99.45**	**99.30**

Respected reviewer,

Thank you for your insightful comment regarding the computational complexity of our proposed algorithm. In the revised manuscript, we have included a detailed discussion outlining the computational complexity of our algorithm and have compared it to existing methods. This comparison sheds light on the efficiency and computational

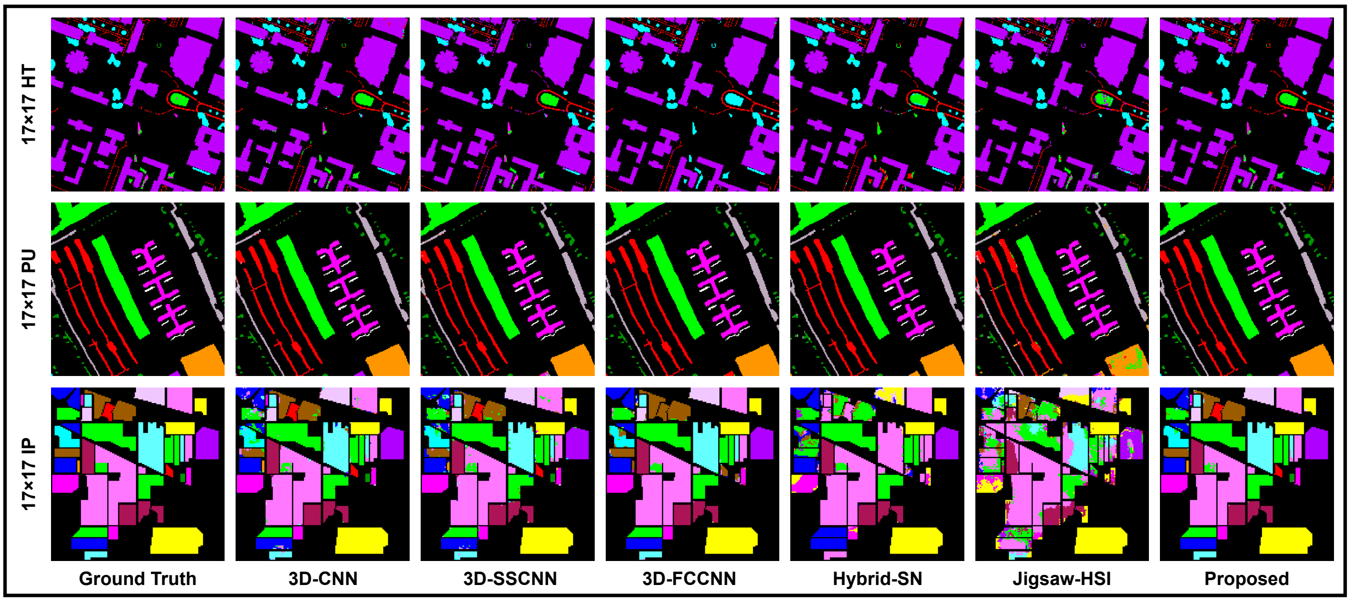

Figure 2: Our models, which incorporate state-of-the-art techniques, perform sophisticated processing of the Ground Truths with high precision and accuracy, considering the complexity of each spatial dimension.

---

advantages of our proposed approach.

Computational efficiency

Each model's performance is measured to determine the computational efficiency. The average run time is presented in Table 6, which shows that information is processed quickly with a small patch size, i.e., 13×13. HybridSN produced the results very fast and placed at the top. 3D-FCCNN is the second-best model to process the model, while Jigsaw-HSI is placed at $3rd$ in this series. Of course, our model is placed as the $4th$ best efficient model. The proposed method is deeper than the comparison algorithm. Our method handles the detailed intrinsic information and provides the best classification results. The proposed model becomes efficient when we go to the larger patch size of 25×25. 3DCDCN predicted the results just in 4.38 seconds, which is the second-best model on the larger size. While the other models cannot manage sustainability. So, we can say that the proposed model is the best fit and reduces the complexity of the HSIs.

Concern # 8

For evaluation purposes, the text should include a discussion section that compares the results with existing literature.

Respected reviewer,
We appreciate your attention to the discussion section. In the updated version, we have incorporated the section titled "Overall Discussion." This section extensively

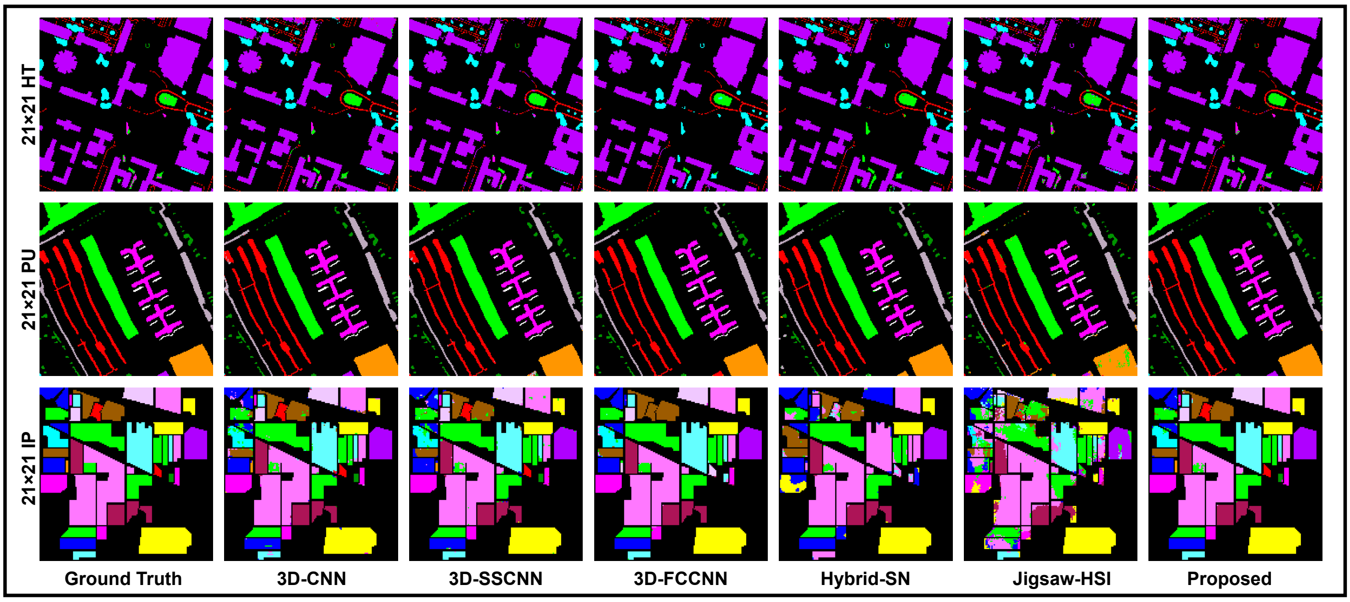

Figure 3: Our models, which incorporate state-of-the-art techniques, perform sophisticated processing of the Ground Truths with high precision and accuracy, considering the complexity of each spatial dimension.

Table 6: The computational times measured in seconds were recorded for the **HT** experimental dataset across various window sizes.

	Window size			
Models	13 × 13	17 × 17	21 × 21	25 × 25
Proposed	1.82	2.01	2.42	4.38
Jigsaw-HSI	1.67	1.95	5.85	9.45
Hybrid-SN	1.15	1.89	1.97	2.58
3D-FCCNN	1.31	1.61	2.31	3.94
3D-SSCNN	1.83	1.42	1.88	2.54
3D-CNN	1.69	1.31	1.71	2.19

covers all experiments conducted in our study, providing a comprehensive and holistic view for readers. Specifically, it elucidates the intricacies of each experiment, contributing to a broader understanding of the manuscript's findings. This inclusive discussion aims to offer readers a detailed and insightful analysis, ensuring they gain a thorough understanding of the research outcomes in the context of the broader scope of the study.

Discussion

Various experiments were performed to measure the effectiveness of the proposed 3D-CDCN attention-dense net. Comprehensive experiments were performed for this purpose. Three publicly available datasets, i.e., HT, PU, and IP, were taken for the experiments. The proposed method was tested on different patch sizes. First, the experiments were performed on the proposed method using different patch sizes. For this purpose, the window size was set to $13 \times 13, 17 \times 17, 21 \times 21$ and 25×25 with

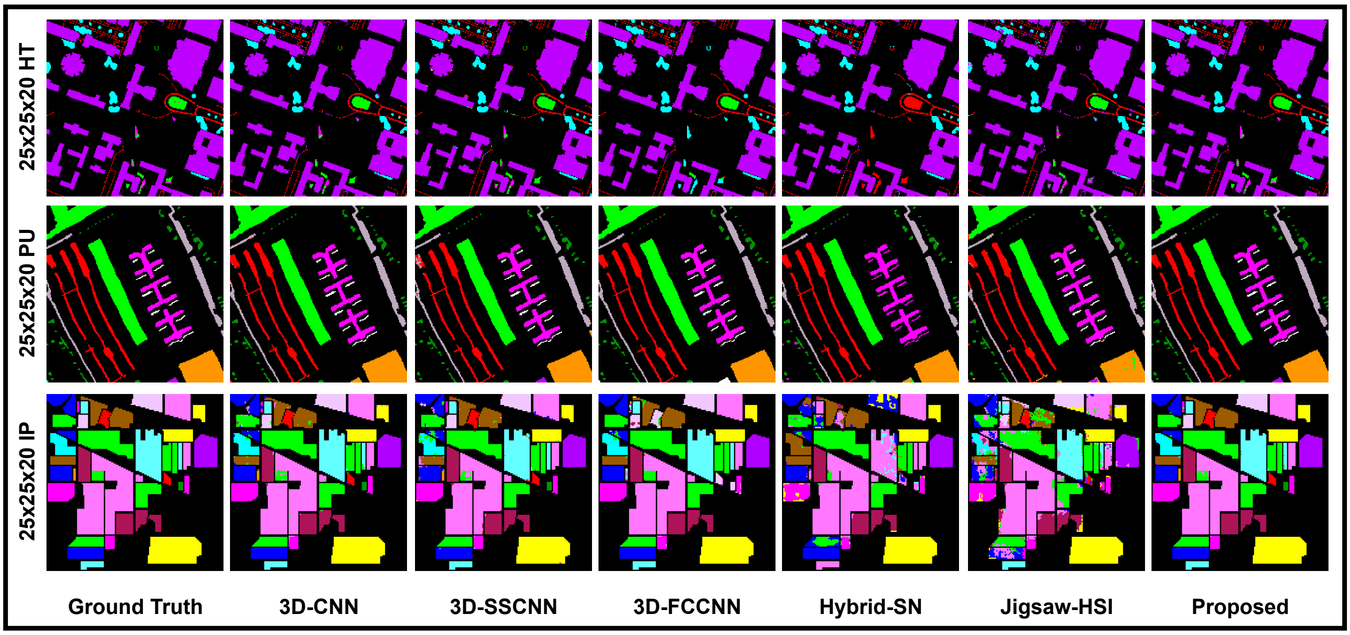

Figure 4: Our models, which incorporate state-of-the-art techniques, perform sophisticated processing of the Ground Truths with high precision and accuracy, considering the complexity of each spatial dimension.

---

24 spectral bands, from the result Table 1 it is noticed that the proposed method's Performance increased with larger patch size.

In the second experiment, we compared the performance of the proposed method with the existing CNN-based methods for a fair comparison. Most methods are 3D, and an equal experimental environment is provided. These methods were also tested on different patch sizes. When we summarized all the experiments of comparing methods, the performance of these methods varied with different patch sizes and on different datasets. For example, the 3D-FCNN produced OA=98.36% on the HT dataset, the second-best result. This model cannot maintain its position on the PU dataset, achieving OA=95.21% and placed at the 3rd position, the same as with the IP dataset. Jigsaw-HSI produced the lowest results with window size 13×13. On the other side, when we see the performance of the proposed 3D-CDCN attention Dense Net with different window sizes, it shows the highest results and maintains its consistency.

The second discussion point is each method's efficiency and capacity. For instance The semi-supervised 3D-CNN can capture distinct spatial classes across various wavelengths, enabling the analysis of a broad spectrum of spectral data. Compared to conventional 2D-CNN, the Spectral-Spatial 3D-SSCNN introduces higher computational complexity and challenges obtaining a diverse and representative HSI dataset for training. However, the Fast and Compact 3D-FCCNN approach has a drawback. It divides the HSI cube into smaller overlapping patches, which leads to a loss of spatial context. Consequently, this method becomes unable to capture global relationships between different regions. The Hybrid-SN and Jigsaw-HSI consider the importance of

interpretability and transparency losses. To address these challenges and improve HSI classification performance and robustness, the clever 3D-CDC Attention DenseNet has been developed. This model focuses on extracting spatial-spectral feature maps, utilizing joint local intrinsic detailed patterns and interrelation among spectral features. The attention mechanism and dense network incorporate low-rank frequency feature information and guide feature tuning. As a result, these advancements have successfully overcome the challenges faced by existing state-of-the-art models.

Concern # 9

This article lacks a background introduction to such massive data. Writing background refers to the historical background in which the author created this article and the context in which it was created. Therefore, please optimize the writing background of the article so that it can have a deeper understanding of the content.

Respected reviewer,
your concerns 1 and 9 are similar. However we have addressed your concern in 1, thank you for your feedback.

Concern # 10

The conclusion of this paper still provides a lot of background information, which obviously does not meet the requirements of conclusion writing. In addition, this article did not explain the shortcomings of the experimental section and the direction of future research.

Respected reviewer,
Thank you for your insightful comments regarding the conclusion section. In response, we have revised the conclusion to focus on summarizing the key findings and insights derived from the study, ensuring it aligns more closely with the requirements of conclusion writing. Additionally, we have incorporated discussions on the shortcomings identified in the experimental section and have provided a clear direction for future research endeavors. This updated conclusion aims to succinctly summarize the study's outcomes, address experimental limitations, and outline potential avenues for future investigations.

Conclusion

In this article, we proposed a method for HSI classification based on a center difference convolution approach that incorporates central difference into vanilla convolution to enhance its representational characteristics and improve its generalization capacity of the convolutional neural network to extract the detailed intrinsic features for the

most accurate classification task. The proposed method exploits the 3D Attention mechanism to explore the more appropriate features, whereas 3D Central Difference CNN is used to extract the detailed intrinsic features, and the dense connections were employed to improve the robustness of the architecture. Although the 2D-CNN and 3D-CNN-based approaches have been widely used for the HSI classification, they have limitations, i.e., 2D CNN does not simultaneously analyze the spatial and spectral data. In contrast, the 3-D CNN emerges as a superior alternative because precise estimation of HSIC requires considering both spatial and spectral features, however, this method ignores the intrinsic features that are important for the accurate classification of HSIs. Our proposed algorithm achieves superior experimental outcomes on three HSI benchmark datasets HT-2018, PU, and IP, establishing state-of-the-art results with different window sizes i.e. $13 \times 13, 17 \times 17, 21 \times 21$ and 25×25 with 24 spectral bands, 3D-CDC Attention DenseNet, produced the better OA score against the comparing methods on all the three datasets, whereas 3D-CDC Attention DenseNet achieved the highest OA% on $25 \times 25 \times 24$ window-sized patch that shows the efficiency of the proposed method. Our experiments demonstrate that our approach not only surpasses conventional 3D CNN-based models but also shows superiority when compared with the baseline deep learning methods, i.e. ID, CNN, 2D CNN, SSFCN, and GCN, and outperforms state-of-the-art networks on various public benchmarks while maintaining lower complexity. For future work, we intend to introduce more convolutional operators with CNNs to make better generalizations of the CNN-based architectures for the HSI classification.

Reviewer 2

Concern # 1

Several current advanced AI methods should be further discussed and analyzed in remote sensing, e.g., SpectralGPT, 10.1109/TGRS.2023.3279834, 10.1016/j.rse.2023.113856

Respected reviewer, Thank you for your suggestion, in the current version we have discussed on the (SpectralGPT, 10.1109/TGRS.2023.3279834 and the third one is out of scope.)

Advanced techniques within the field of deep learning have been suggested for dealing with the problem of shifting domains in hyperspectral imaging (HSI). The paper [14] introduces a new approach called LRR-Net for anomaly detection. LRR-Net is a baseline network that combines the low-rank representation (LRR) model with deep learning techniques. LRR-Net employs the ADMM optimizer to efficiently solve the LRR model and convert normal parameters into trainable ones, hence reducing the need for human adjustment. The paper [9] presents a novel and comprehensive

framework for remote sensing (RS) applications, specifically designed to overcome the limited emphasis on spectral data in visual representation learning. SpectralGPT is designed specifically for processing spectral remote sensing (RS) images, unlike conventional models that mainly focus on RGB images. It utilizes a 3D-generated pre-trained transformer (GPT) architecture. It distinguishes itself by providing support for images of diverse sizes, resolutions, and time series through incremental training. A spatial and spectral BERT is proposed in [1] utilizing the local and global features to improve the HSI classification.

(Concern # 2)

The reviewer is also wondering about the computational complexity.

Respected reviewer, Please see the part 4.4 (at line number 504)

Computational efficiency

Each model's performance is measured to determine the computational efficiency. The average run time is presented in Table. 8, which shows that information is processed quickly with a small patch size, i.e., 13×13. HybridSN produced the results very fast and placed at the top. 3D-FCCNN is the second-best model to process the model, while Jigsaw-HSI is placed at $3rd$ in this series. Of course, our model is placed as the $4th$ best efficient model. The proposed method is deeper than the comparison algorithm. Our method handles the detailed intrinsic information and provides the best classification results. The proposed model becomes efficient when we go to the larger patch size of 25×25. 3DCDCN predicted the results just in 4.38 seconds, which is the second-best model on the larger size. While the other models cannot manage sustainability. So, we can say that the proposed model is the best fit and reduces the complexity of the HSIs.

Reviewer 4

Concern # 1

The authors addressed all my previous comments, I recommend the revised manuscript for possible publication.

Respected reviewer, Thank you very much for acknowledging our work

Reviewer 5

Concern # 1

Comparison with base-line deep learning method must be presented.

Respected reviewer,

Comparsion with base-line deep learning methods

We have extensively evaluated the performance of the proposed approach, which is composed of a 3D layered architecture, by comparing it to several deep learning methods. At first, we examined our method in the context of 3D layered architecture during the experimental phase. Afterward, we expanded our research to include deep learning methods that expand beyond this specific architecture. The provided comparison table in this part clearly shows the superiority of the approach we propose compared to common deep learning models such as 1D CNN, 2D-CNN, SSFCN, and GCN across three distinct datasets: HT, PU, and IP. The 1D Convolutional Neural Network (CNN) has a reasonable level of performance, with the maximum overall accuracy (OA) of 80.23% in the HT dataset. Its performance does, however, significantly decline in the PU and IP datasets, indicating there are limitations when handling these kinds of data. While 2D-CNN outperforms 1D CNN, particularly in the PU dataset with an overall accuracy (OA) of 88.19%, it is still not as effective as more advanced models. This implies that its ability to capture the complex structure of the datasets is limited. The SSFCN model consistently achieves good performance across all datasets, with its maximum overall accuracy (OA) of 89.62% observed in the IP dataset. This demonstrates a superior ability to manage diverse data structures in contrast to the prior models. GCN exhibits better performance on the HT dataset with an Overall Accuracy (OA) of 71.41%, its effectiveness significantly diminishes in the IP dataset, indicating possible challenges in extrapolating results across heterogeneous data types. The proposed method, demonstrates superior performance compared to all other models across all datasets, with outstanding Overall Accuracy (OA) scores of 97.93%, 99.89%, and 99.3% in the HT, PU, and IP datasets, respectively. The good results show the efficacy of the 3D layered architecture in managing diverse and intricate data structures. The significant superiority of our method over the baseline models emphasizes its improved capacity to reliably identify and predict results, even in complex scenarios.

The investigation highlights the improved efficiency of the proposed method in handling complex data structures and its superiority over conventional deep learning models. This comparison analysis not only confirms the success of our approach but also provides the way for its implementation in increasingly complex and diverse based on data environments.

Concern # 2

The proposed research must be evaluated on a large scale dataset otherwise mentioned a reason.

Table 7: Comparison with base-line deep learning methods

Datasets	HT			PU			IP		
Models	OA	AA	Kappa	OA	AA	Kappa	OA	AA	Kappa
1D CNN	80.23	82.09	78.61	75.39	83.13	68.73	73.19	83.29	69.52
2D-CNN	84.08	87.01	82.77	88.19	89.98	84.49	82.94	92.88	80.55
SSFCN	84.14	85.84	82.86	85.99	90.32	81.83	89.62	94.25	88.12
GCN	85.73	86.41	84.54	81.83	87.60	76.75	70.22	79.00	66.27
Proposed	**97.93**	**71.74**	**92.80**	**99.89**	**88.82**	**99.86**	**99.38**	**99.45**	**99.30**

Respected reviewer,Thank you for your valuable feedback regarding the evaluation of our research on a larger scale dataset. We understand the importance of validating models on extensive datasets to ensure generalizability and robustness. In our study, we utilized the Houston-2018, Pavia University, and Indian Pines datasets for the following reasons:

1. Representative Diversity: These datasets are widely recognized in the hyperspectral image classification community and offer a diverse range of spectral signatures and land cover classes. This diversity is critical for testing the efficacy of our proposed method across different types of hyperspectral imagery.

2. Benchmarking Standards: These datasets serve as standard benchmarks in the field, allowing for direct comparison with prior works. Using these datasets ensures that our results are comparable and relevant to ongoing research.

3. Limitations in Availability of Larger Datasets: While larger-scale. datasets could potentially provide a more comprehensive evaluation, there is a notable scarcity of publicly available, large-scale hyperspectral datasets. This limitation is a recognized challenge in the field of hyperspectral image classification.

4. Preliminary Nature of Research: Our study is exploratory in nature, aiming to establish foundational insights and methodologies. The chosen datasets are appropriate for this stage, providing a balanced approach between complexity and manageability.

Concern # 3

The mathematical model is not defined well, a detailed explanation is required.

We are thankful for your feedback and appreciate your comments. Respected reviewer, In the revised version we have included the possible explanation of the mathematical model in the section 3.1.4

Detailed Derivation for CDC

We have thoroughly examined the precise derivation of the Central Difference Convolution (CDC), which is a crucial element of our suggested model. This derivation seeks to clarify the mathematical foundations of the CDC, including its essential elements, the Central Difference Term (CDT) and Vanilla Convolution (VC). The

elements stated combined provide the key basis of our method, as represented by Equation 7. This discussion aims to clarify the operational mechanics of the CDC, offering a clear understanding of how it significantly improves the feature extraction capabilities of our model.

$$
\begin{aligned}
Y(P_0) = \theta_h \cdot & \underbrace{\sum_{P_n \in R_l} W_{sw}(P_n) \cdot (X_{fm}(P_0 + P_n) - X_{fm}(P_0))}_{\text{CDC}} \\
& + (1 - \theta_h) \cdot \underbrace{\sum_{P_n \in R_l} W_{sw}(P_n) \cdot X_{fm}(P_0 + P_n)}_{\text{VC}} \\
& = \theta_h \cdot \underbrace{\sum_{P_n \in R_l} W_{sw}(P_n) \cdot (X_{fm}(P_0 + P_n)}_{\text{VC}} \\
& + \theta_h \cdot (- \underbrace{\sum_{P_n \in R_l} W_{sw}(P_n) \cdot (X_{fm}(P_0))}_{\text{CDT}} \\
& + (1 - \theta_h) \cdot \underbrace{\sum_{P_n \in R_l} W_{sw}(P_n) \cdot (X_{fm}(P_0 + P_n)}_{\text{VC}} \\
& = (\theta_h + 1 - \theta_h) \cdot \underbrace{\sum_{P_n \in R_l} W_{sw}(P_n) \cdot (X_{fm}(P_0 + P_n)}_{\text{VC}} \\
& + \theta_h \cdot (-X_{fm}(P_0) \cdot \underbrace{\sum_{P_n \in R_l} W_{sw}(P_n))}_{\text{CDT}} \\
& = \theta_h \cdot \underbrace{\sum_{P_n \in R_l} W_{sw}(P_n) \cdot (X_{fm}(P_0 + P_n)}_{\text{VC}} \\
& + \theta_h \cdot (-X_{sw}(P_0) \cdot \underbrace{\sum_{P_n \in R_l} W_{sw}(P_n)}_{\text{CDT}}
\end{aligned} \tag{4}
$$

In this equation, $Y(P_0)$ represents the output at the central pixel P_0. θ_h is a hyperparameter that balances the contributions of the central difference term (CDC) and the value convolution term (VC). $W_{sw}(P_n)$ denotes the spatial weights for neighboring pixels P_n within the local region R_l. $X_fm(P_0 + P_n)$ and $X_fm(P_0)$ are the feature map values at the neighboring pixel P_n and the central pixel P_0 , respectively. The model combines the CDC and VC terms, where the CDC captures local differences in feature maps, emphasizing edges and fine details. At the same time, the VC aggre-

gates local information, ensuring robustness to noise. The hyperparameter θ_h allows for the adjustment of the model's sensitivity to local differences.

Concern # 4

The details about fine-tunned parameters/optimization details are missing.

We are thankful for your feedback and appreciate your comments. Respected reviewer, we have already discussed fine-tunned parameters/ optimization detail, we have highlighted this detail in magenta color in the manuscript

Experimental Settings

Experiments are done in this study using the proposed model and comparing methods. Three well-known HS HSI datasets were taken to check the efficiency of the 3DCDCN. Houston-2018 (HT), IP, and PU were used to test. All the experiments were done in Colab Pro. In all experiments, the initial Test/Train set was divided into a 30 : 70, ratio, with 70% of the population reserved for train samples and the remaining 30% for test samples. To ensure fair evaluations, we used a standardized learning rate of $(1e-02)$ and a decay rate of $(1e-06)$ for all experiments. Furthermore, we utilized the rectified linear unit activation function in all layers except for the last layer, where we employed the softmax activation. The patch sizes used in our experiments were $13 \times 13 \times 20, 17 \times 17 \times 20, 21 \times 21 \times 20$, and $25 \times 25 \times 20$, respectively. These patch sizes were determined using the PCA method to identify the 20 most informative bands. For optimization, the Adam optimizer was used, and 100 epochs were set to train the model.

Reviewer 6

Concern # 1

Discourage the usage of words 'we', 'our', and 'they'.

Respected reviewer, Thank you for providing the guideline to improve the article. We have revised the article, and removed all the possible words 'we', 'our', and 'they'

(Concern # 2)

Authors need to add a result line in the Abstract.

Respected reviewer, Thank you for your suggestion, in the revised version we have included the result line in the abstract, we have included these lines in the abstract.

" The proposed method achieved 97.93% overall accuracy on the Houston-2018,

99.89% on Pavia University and showed the 99.38% on the Indian Pines dataset with the 25×25 window size"

Concern # 3

Please confirm Figure 1 you drew or add a proper citation to it.

Respected reviewer, We appreciate your concern related to figure 1; it is drawn by our self.

Concern # 4
There are some typos in the paper. Authors need to carefully re-write the long sentences of the paper.

Respected Reviewer, We appreciate your critical analysis towards our article, we did multiple revisions, and we did our best to improve the quality of the paper.

References

[1] Mahmood Ashraf, Xichuan Zhou, Gemine Vivone, Lihui Chen, Rong Chen, and Reza Seifi Majdard. Spatial-spectral bert for hyperspectral image classification. *Remote Sensing*, 16(3):539, 2024.

[2] Yushi Chen, Xing Zhao, and Xiuping Jia. Spectral–spatial classification of hyperspectral data based on deep belief network. *IEEE journal of selected topics in applied earth observations and remote sensing*, 8(6):2381–2392, 2015.

[3] Yushi Chen, Lin Zhu, Pedram Ghamisi, Xiuping Jia, Guoyu Li, and Liang Tang. Hyperspectral images classification with gabor filtering and convolutional neural network. *IEEE Geoscience and Remote Sensing Letters*, 14(12):2355–2359, 2017.

[4] Random Fields. Hyperspectral image classification with markov random fields and a convolutional neural network. *learning*, 19:42.

[5] Dren Gashi, Mike Pereira, and Valeriia Vterkovska. Multi-scale context aggregation by dilated convolutions machine learning-project. 2017.

[6] Robert O Green, Michael L Eastwood, Charles M Sarture, Thomas G Chrien, Mikael Aronsson, Bruce J Chippendale, Jessica A Faust, Betina E Pavri, Christopher J Chovit, Manuel Solis, et al. Imaging spectroscopy and the airborne visible/infrared imaging spectrometer (aviris). *Remote sensing of environment*, 65(3):227–248, 1998.

[7] Amina Ben Hamida, Alexandre Benoit, Patrick Lambert, and Chokri Ben Amar. 3-d deep learning approach for remote sensing image classification. *IEEE Transactions on geoscience and remote sensing*, 56(8):4420–4434, 2018.

[8] Danfeng Hong, Naoto Yokoya, Jocelyn Chanussot, and Xiao Xiang Zhu. An augmented linear mixing model to address spectral variability for hyperspectral unmixing. *IEEE Transactions on Image Processing*, 28(4):1923–1938, 2018.

[9] Danfeng Hong, Bing Zhang, Xuyang Li, Yuxuan Li, Chenyu Li, Jing Yao, Naoto Yokoya, Hao Li, Xiuping Jia, Antonio Plaza, et al. Spectralgpt: Spectral foundation model. *arXiv preprint arXiv:2311.07113*, 2023.

[10] Wei Hu, Yangyu Huang, Li Wei, Fan Zhang, and Hengchao Li. Deep convolutional neural networks for hyperspectral image classification. *Journal of Sensors*, 2015:1–12, 2015.

[11] Felix Juefei-Xu, Vishnu Naresh Boddeti, and Marios Savvides. Local binary convolutional neural networks. In *Proceedings of the IEEE conference on computer vision and pattern recognition*, pages 19–28, 2017.

[12] B Kunkel, F Blechinger, R Lutz, R Doerffer, H Van der Piepen, and M Schroder. Rosis (reflective optics system imaging spectrometer)-a candidate instrument for polar platform missions. In *Optoelectronic technologies for remote sensing from space*, volume 868, pages 134–141. SPIE, 1988.

[13] Hyungtae Lee and Heesung Kwon. Going deeper with contextual cnn for hyperspectral image classification. *IEEE Transactions on Image Processing*, 26(10):4843–4855, 2017.

[14] Chenyu Li, Bing Zhang, Danfeng Hong, Jing Yao, and Jocelyn Chanussot. Lrr-net: An interpretable deep unfolding network for hyperspectral anomaly detection. *IEEE Transactions on Geoscience and Remote Sensing*, 2023.

[15] Wei Li, Chen Chen, Hongjun Su, and Qian Du. Local binary patterns and extreme learning machine for hyperspectral imagery classification. *IEEE Transactions on Geoscience and Remote Sensing*, 53(7):3681–3693, 2015.

[16] Wei Li, Guodong Wu, Fan Zhang, and Qian Du. Hyperspectral image classification using deep pixel-pair features. *IEEE Transactions on Geoscience and Remote Sensing*, 55(2):844–853, 2016.

[17] Min Lin, Qiang Chen, and Shuicheng Yan. Network in network. *arXiv preprint arXiv:1312.4400*, 2013.

[18] Zhouhan Lin, Yushi Chen, Xing Zhao, and Gang Wang. Spectral-spatial classification of hyperspectral image using autoencoders. In *2013 9th international*

conference on information, Communications & Signal Processing, pages 1–5. IEEE, 2013.

[19] Shangzhen Luan, Chen Chen, Baochang Zhang, Jungong Han, and Jianzhuang Liu. Gabor convolutional networks. *IEEE Transactions on Image Processing*, 27(9):4357–4366, 2018.

[20] ME Midhun, Sarath R Nair, VT Nidhin Prabhakar, and S Sachin Kumar. Deep model for classification of hyperspectral image using restricted boltzmann machine. In *Proceedings of the 2014 international conference on interdisciplinary advances in applied computing*, pages 1–7, 2014.

[21] Jiquan Ngiam, Zhenghao Chen, Daniel Chia, Pang Koh, Quoc Le, and Andrew Ng. Tiled convolutional neural networks. *Advances in neural information processing systems*, 23, 2010.

[22] Sakrapee Paisitkriangkrai, Jamie Sherrah, Pranam Janney, Van-Den Hengel, et al. Effective semantic pixel labelling with convolutional networks and conditional random fields. In *Proceedings of the IEEE Conference on Computer Vision and Pattern Recognition Workshops*, pages 36–43, 2015.

[23] Hyperspectral Remote Sensing Scenes. Available online: http://www.ehu.eus/ccwintco/index.php. *Hyperspectral_Remote_Sensing_Scenes (accessed on 22 April 2020)*, 2020.

[24] Christian Szegedy, Wei Liu, Yangqing Jia, Pierre Sermanet, Scott Reed, Dragomir Anguelov, Dumitru Erhan, Vincent Vanhoucke, and Andrew Rabinovich. Going deeper with convolutions. In *Proceedings of the IEEE conference on computer vision and pattern recognition*, pages 1–9, 2015.

[25] Qi Wang, Zhenghang Yuan, Qian Du, and Xuelong Li. Getnet: A general end-to-end 2-d cnn framework for hyperspectral image change detection. *IEEE Transactions on Geoscience and Remote Sensing*, 57(1):3–13, 2018.

[26] Jun Yue, Wenzhi Zhao, Shanjun Mao, and Hui Liu. Spectral–spatial classification of hyperspectral images using deep convolutional neural networks. *Remote Sensing Letters*, 6(6):468–477, 2015.

[27] Haokui Zhang, Ying Li, Yuzhu Zhang, and Qiang Shen. Spectral-spatial classification of hyperspectral imagery using a dual-channel convolutional neural network. *Remote sensing letters*, 8(5):438–447, 2017.

[28] Jian Zhu, Leyuan Fang, and Pedram Ghamisi. Deformable convolutional neural networks for hyperspectral image classification. *IEEE Geoscience and Remote Sensing Letters*, 15(8):1254–1258, 2018.
