# Supplementary material for: Attention 3D central difference convolutional dense network for hyperspectral image classification
Source: PLoS One. 2024 Apr 10;19(4):e0300013. doi: 10.1371/journal.pone.0300013 (PMC11006129; doi:10.1371/journal.pone.0300013)
Supplement: Attachment — Submitted filename: Rebuttal Letter for PLOS.docx [file pone.0300013.s002.docx]

**Rebuttal Letter for: 3D Central Difference Convolutional Dense Network for Hyperspectral Image Classification**

We would like to thank the editors and the reviewers for their comments. In this rebuttal letter, we addressed all the comments/suggestions. The revised content and the related position in the paper are detailed in the point-by-point reply. Noteworthy, in the point-by-point reply, the reviewers’ comments are in green italic. Instead, the changes that have been performed in the revised manuscript are in blue.

**Reviewer #1:**

Your research addresses an important issue, and your proposed algorithm shows promise. However, there are several areas that could be improved to enhance the clarity and rigor of your work. Here are the specific suggestions for revision:

*1. This article lacks a background introduction to such massive data. Writing background refers to the historical background in which the author created this article, and the context in which it was created. Therefore, please optimize the writing background of the article so that it can have a deeper understanding of the content*.

**Response to Reviewer#1:** We sincerely thank you for guiding us to enrich the manuscript's content. The revisions made have significantly bolstered the depth and relevance of the literature review, ensuring that it adequately reflects the advancements in the field.

*2. In the introduction section of the article, the author's expression of the writing meaning is somewhat vague and difficult to understand. Please reorganize the language to express it so that readers can quickly understand the writing meaning and purpose of the article.*

**Response to Reviewer#1:** Thank you for your valuable suggestion to enhance the introduction section. We've revised it by simplifying the language and replacing complex words with simpler ones. As a result, the updated introduction is now clearer and more easily understandable.

*3.* *This article introduces the research of multiple scholars and explains their research methods. However, the literature section can emphasize the shortcomings of various methods, which is enough to shift to newly proposed methods.*

**Response to Reviewer#1:** Thank you for your feedback. We've meticulously highlighted the limitations of existing methods in the literature section to justify the introduction of our novel approaches. The discussion emphasizes these drawbacks as a basis for proposing our innovative methodologies, aiming to address these identified shortcomings. We've ensured a clear transition, illustrating how the proposed methods offer improvements over established techniques

*4. Provide information on the specific data used in your study. Mention the source and characteristics of the data.*

**Response to Reviewer#1:** Thank you for your feedback. In the updated version, we've incorporated the data source and provided a concise discussion on the dataset.

*6. Describe the simulation environment and parameters used for testing the algorithm.*

**Response to Reviewer#1:** Thank you for your valuable feedback regarding the simulation environment and parameters utilized in this study. In response, we have implemented a dedicated section titled "Experimental Settings," wherein we comprehensively list and detail all the training and testing parameters employed in our experiments.

*7. Include a discussion on the metrics used to evaluate the algorithm's performance and why these metrics were chosen.*

**Response to Reviewer#1:** We appreciate your suggestion regarding the inclusion of a discussion on the evaluation metrics used to assess the algorithm's performance. In the updated version, we've incorporated a detailed section discussing the chosen evaluation metrics and the rationale behind their selection.

*8. Provide a more detailed presentation of the simulation results, including tables or graphs to illustrate the improvements achieved.*

**Response to Reviewer#1:** Thank you for your valuable feedback regarding the presentation of simulation results. In response, we have enhanced the manuscript by providing a more detailed presentation of the simulation outcomes. We have included all window sized maps of all the models more discussion on the experiments are included in the current version and to vividly illustrate the improvements achieved by our proposed approach.

*9. Discuss the computational complexity of your proposed algorithm and how it compares to existing methods.*

**Response to Reviewer#1:** Thank you for your insightful comment regarding the computational complexity of our proposed algorithm. In the revised manuscript, we have included a detailed discussion outlining the computational complexity of our algorithm and have compared it to existing methods. This comparison sheds light on the efficiency and computational advantages of our proposed approach

*10. For evaluation purposes, the text should include a discussion section that compares the results with existing literature.*

**Response to Reviewer#1:** We appreciate your attention to the discussion section. In the updated version, we have incorporated the section titled "Overall Discussion." This section extensively covers all experiments conducted in our study, providing a comprehensive and holistic view for readers. Specifically, it elucidates the intricacies of each experiment, contributing to a broader understanding of the manuscript's findings. This inclusive discussion aims to offer readers a detailed and insightful analysis, ensuring they gain a thorough understanding of the research outcomes in the context of the broader scope of the study.

*11. What are the limitations of this study?*

**Response to Reviewer #1**: Thank you for your inquiry regarding the limitations of our study. Several constraints are present within our research that merit acknowledgment. Firstly, our study's findings might be influenced by the characteristics and size of the dataset used, potentially impacting the model's generalizability. Additionally, methodological constraints and computational limitations might have affected the depth and breadth of our analysis, potentially restricting the scope of our conclusions. We also recognize that our study might have limitations regarding external validity, as our findings may not directly apply to all real-world scenarios or domains due to constraints in experimental settings or data availability. Furthermore, assumptions made during the study and any temporal or scope constraints may have influenced the interpretation and applicability of our results. However, we have added a separate section titled “Limitation of the network”.

*12. The conclusion of this paper still provides a lot of background information, which obviously does not meet the requirements of conclusion writing. In addition, this article did not explain the shortcomings of the experimental section and the direction of future research.*

**Response to Reviewer #1:** Thank you for your insightful comments regarding the conclusion section. In response, we have revised the conclusion to focus on summarizing the key findings and insights derived from the study, ensuring it aligns more closely with the requirements of conclusion writing. Additionally, we have incorporated discussions on the shortcomings identified in the experimental section and have provided a clear direction for future research endeavors. This updated conclusion aims to succinctly summarize the study's outcomes, address experimental limitations, and outline potential avenues for future investigations.

**Reviewer #2:**

 This paper proposed an Attention 3D Central Difference Convolutional Dense Network for Hyperspectral Image Classification. Overall, the structure of this paper is well organized, and the presentation is clear. However, there are still some crucial problems that need to be carefully addressed before a possible publication. More specifically,

*1. A deep literature review should be given, particularly advanced and SOTA deep learning or AI models in remote sensing*

**Response to Reviewer#2:** We sincerely thank you for guiding us to enrich the manuscript's content. The revisions made have significantly bolstered the depth and relevance of the literature review, ensuring that it adequately reflects the advancements in the field. Moreover, we have separately added the related work in the updated version.

*2. Please clarify the contributions, and what are the newly-added values compared to the existing methods.*

**Response to Reviewer#2:** Thank you for your query regarding the contributions and the unique values introduced in our study compared to existing methods. Our research contributes to the field by

1- As of our knowledge we are the first who introduced CDCN model in HSI domain, we introduced 3D CDCN
2- We introduced the Spatial attention module with this network as in the figure :1
3- We introduced dense connection instead of residual connection with the proposed network

The newly added values compared to the existing method lie in the ablation study and the improvement with novel modules can be seen in Table 6. Specifically, our method simultaneously processes the spatial and spectral dimensions of the HS data. Distinguishes it from prior approaches in classifying the HSI data with the highest accuracy

*3. The workflow should be drawn to clarify the proposed method.*

**Response to Reviewer#2:** Thank you for your suggestion regarding the need for the proposed method's workflow. In the revised manuscript, we have included a detailed workflow as presented in Figure 1. We also improved the module-wise explanation in the revised version.

*4. The ablation analysis should show the performance gain from newly-added modules.*

**Response to Reviewer#2:** Thank you for your suggestion regarding the ablation analysis. In the revised manuscript, we have conducted an extensive ablation study to demonstrate the performance gain achieved from the newly added modules in our proposed method. Specifically, we have systematically evaluated the impact of each newly introduced module by selectively removing or deactivating them from the model and analyzing the corresponding performance changes. This analysis provides valuable insights into the contribution of these modules to the overall performance improvement

*5. How about the computational complexity?*

Thank you for inquiring about the computational complexity of our study. In the revised version we have discussed the computational complexities of the proposed model and the comparing methods in the “Model efficiency” subsection. The proposed model is based on 3D and equipped with dense modules. Not more efficient in time, but producing good classified results, that can be acceptable with good accuracy.

**Response to Reviewer#2:** Thank you for your interest in the computational complexity within our study. In the revised version, we've delved into the computational intricacies of both the proposed model and the comparative methods within the "Model Efficiency" subsection. Our proposed model, designed with 3D architecture and incorporating a dense module, demonstrates good classification results, offering the highest accuracy despite being relatively less time-efficient.

6. It is well-known that the remote sensing data usually tend to suffer from various degradation, noise effects, or variabilities in the process of imaging. Please give the discussion and analysis by referring to the paper titled by e.g., An Augmented Linear Mixing Model to Address Spectral Variability for hyperspectral unmixing. The reviewer is wondering what will happen if the proposed method meets the various variabilities.

**Response to Reviewer#2:** First of all, thank you so much for sharing this precious research work is with us that resolves an important aspect of HSI classification and denoising. We have cited this research work in the related work section to improve our related work with the following lines: “In a study, the augmented linear mixing model (ALMM) tackles spectral variability in hyperspectral images by using a data-driven approach to isolate scaling factors linked to illumination or typography using an endmember dictionary, while also capturing additional variations from environmental factors and instrument settings via spectral variability dictionary [39]. Their proposed method, integrated into the spectral unmixing framework, allows for the concurrent acquisition of the spectral variability dictionary and the estimation of abundance maps, showcasing enhanced effectiveness compared to earlier cutting-edge techniques in experiments conducted on both synthetic and real datasets.”

Respected reviewer, this research work is part of my PH.D. and at the moment my topic is more related to HSI classification with higher accuracy rate. The usage of spectral variability approach is a different and optimized domain in HSI classification that surely, I want to pursue during my PostDoc. Some times usage of spectral variability approaches leads to less and inaccurate results. Some of the reasons are:

1. Homogeneous Regions: In cases where the hyperspectral image predominantly contains homogeneous regions with minimal spectral variations, calculating spectral variability may not provide meaningful insights. If the entire scene or most of the objects have similar spectral characteristics, the spectral variability may not contribute significantly to distinguishing features.
2. Noise Dominance: If the hyperspectral data is noisy, and the noise level is comparable to or higher than the actual spectral variations due to different materials or objects, relying on spectral variability might lead to inaccurate or unreliable results. In such situations, noise reduction techniques may be more critical than spectral variability analysis.
3. Simple Classification Tasks: For straightforward classification tasks where the classes are easily separable based on a few dominant spectral features, employing spectral variability may be unnecessary and could add complexity without significant benefit.

*7. Some future points should be pointed out in the conclusion.*

**Response to Reviewer#2:** Appreciation for your insightful input concerning the conclusion section, In the revised manuscript, we have expanded the conclusion to include key points highlighting potential future directions for research in this field. These future considerations encompass to introduce more convolutional operators with CNNs to make better generalizations of the CNN-based architectures for the HSI classification. By incorporating these points in the conclusion, we aim to provide valuable insights into potential avenues for further investigation and advancement within this domain.

**Reviewer #3:**

This article introduces a novel approach called the Attention 3D Central Difference Convolutional Dense Network. It uses the 3D-Central Difference Convolutional method to handle local intrinsic patterns in the spatial-spectral feature maps. This approach incorporates pixel-level concatenation and spatial attention mechanism within a dense strategy to include low-rank frequency features and guide feature turning. Although this idea is helpful for Hyperspectral image classification, there are still some issues that need to be addressed.

*1、Please provide the abbreviations that appear in the manuscript along with their corresponding full forms. For example, 3D-CDC.*

**Response to Reviewer#3:** Thank you very much for guiding us to improve the quality of the manuscript. In the updated version we have provided the abbreviation along with their full name in Table 1.

3D-CDC = 3D central difference convolution

3D-CDCN = 3D central difference convolutional neural network

CDCN = central difference convolutional neural network

HSI = Hyperspectral image classification

HS image= Hyperspectral images

SA= spatial Attention

CNN= Convolutional neural network.

2、Please adjust the placement of the citation in the text, for example, in line 60.

**Response to Reviewer#3:** Thank you for your valuable comment. We have corrected the placement of citations.

3、*Please specify the content corresponding to each chapter before the Proposed Methodology.*

**Response to Reviewer#3:** Thank you for your valuable input. In response to your request, we have outlined the content of each chapter preceding the Proposed Methodology section. Before starting the methodology, at the end of the introduction part we have already provided the chapter-wise description, more over we have highlighted it with the color.

4、Please provide the figures mentioned in the manuscript at the title position of the figures section.

**Response to Reviewer#3:** Thank you for your valuable input. In response to your request, we have placed figure right above the figure caption. In first submission, we have separately provided the figures due to journal requirements.

5、Please provide the standardized formatting for Table 1, Table 2, Table 3, Table 4, and Table5 in the manuscript.

**Response to Reviewer#3:** Thank you for your valuable input. We have carefully formatted all the tables according to Plos One published paper format. All these tables are composed in latex if any small format mistake left it will be covered by Plos one publication department.

6、Please standardize the font size of the title in the manuscript.

**Response to Reviewer#3:** Respected reviewer, we have used Plos One latex format. In this latex file, each section of title, abstract, and subsections are defined by using predefined commands. We have not changed anything. We submitted and published 3 other papers using same format. Minor formatting done by Plos One publication department.

*7、Please provide a clear description of the Central Difference Convolution module mentioned in the manuscript*.

**Response to Reviewer#3:** Thank you for your inquiry regarding the Central Difference Convolution (CDCN) module employed in our manuscript.

The CDCN module, as utilized in our research, comprises a convolutional layer designed to extract features from hyperspectral data. Specifically, it leverages the concept of central differences within the convolutional operation to capture intricate spectral-spatial information present in hyperspectral images.

In essence, the CDCN module computes the central differences between adjacent spectral bands or channels during the convolution process. By utilizing this technique, the module aims to enhance feature representation by emphasizing subtle spectral variations across neighboring bands. This allows for the extraction of discriminative features that encapsulate both spectral and spatial characteristics unique to hyperspectral data.

Our manuscript overview the detailed implementation of the CDCN module within the context of hyperspectral image processing. We provide comprehensive insights into the mathematical formulation and computational intricacies of this module, emphasizing its role in enhancing feature extraction for hyperspectral analysis.

We trust that the revised manuscript offers a clearer description of the CDCN module and its application in hyperspectral data analysis.

*8、Please further expand the experiment content of the network based on the given experiment in the manuscript (e.g., the computational complexity of the network) to fully demonstrate whether the proposed solution in the manuscript is reasonable.*

**Response to Reviewer#3:** We sincerely appreciate your concern related to expansion of the experimental work, in the revised version we have discussed the experiments in more detail in the experimental portion, moreover we have separately discussed the computational aspects of the all the methods, which showing the effectiveness of the proposed methods. Additionally, we conducted a comparative analysis against existing methodologies, highlighting the efficiency gains achieved by our approach without compromising performance

**Reviewer #4:**

The Authors proposed 3D difference Dense NET framework for HSI classification. The performance of the proposed framework experimented on the benchmark datasets. However, the following comments need to address before manuscript accept for publication.

*1. Novelty and contribution needs to specify properly.*

**Response to Reviewer#4:** We deeply value your emphasis on the novelty and contributions of our work. In the revised version, we have meticulously spotlighted the distinctive novelty and substantial contributions of our proposed framework

*2. How proposed framework differ from the existing 3DCNN CDC framework?*

**Response to Reviewer#4:** The proposed framework differs from the existing 3DCNN framework, even though both are applied to hyperspectral images, in the following ways:

1-**Feature Extraction Approach**: The proposed framework might introduce novel feature extraction methods optimized explicitly for hyperspectral data. A specialized technique is utilized (CDC) within the 3DCNN architecture to capture and emphasize spectral-spatial patterns unique to hyperspectral images. This tailored approach could differ significantly from the standard feature extraction methodology in the existing 3DCNN framework.

2-**Model Adaptations for Hyperspectral Analysis:** The proposed framework could incorporate adaptations or modifications within the 3DCNN architecture to better suit hyperspectral image analysis. This might involve specific adjustments in convolutional layers and attention mechanisms to enhance the extraction of spectral features compared to the general-purpose approach of the existing 3DCNN framework.

*3. Literature review weak. Author’s needs to study the recent existing 3DCNN CDC models for HSI classification and Spectral spatial features based HSI classification frameworks and same needs to compare the performance of the proposed.*

**Response to Reviewer#4:**

We appreciate your comment, we have added SOTA 3DCNN CDC models for HSI classification and also compared their performance with the proposed approach.

In the revised version we have expand the introduction part and as well as one more section “related work” included that enhancing the importance of the proposed methods. Moreover our method is 3D-CDCN which is unique in hyperspectral imaging domain, as our knowledge we are the first who introduced this in HSI domain. Our proposed method is 3D, in the comparison most of the methods are 3D.

4. Convergence analysis is missing. Is the parameters used in the framework generalize?

**Response to Reviewer#4:**

We appreciate your comment, Respected reviewer, in section 4.1.1 we have elaborated the parameters we used in the proposed model. In figure 4, we have output of proposed model neurons processing. We have tuned the parameters according to the nature of dataset to obtained optimal results. We also added section 4.3 to elaborate which factors helpful in fast convergence of the model.

5. Justifications for fast convergence is required.

**Response to Reviewer#4:**

We appreciate your comment, Respected reviewer, we also added section 4.3 to elaborate which factors helpful in fast convergence of the model.
